# Initial Inventory of Plastics Imports in Nigeria as a Basis for More Sustainable Management Policies

**DOI:** 10.5696/22156-9614-8.18.1

**Published:** 2018-06-11

**Authors:** Joshua O. Babayemi, Mary B. Ogundiran, Roland Weber, Oladele Osibanjo

**Affiliations:** 1 Department of Chemical and Food Sciences, Bells University of Technology, Ota, Nigeria; 2 Department of Chemistry, Faculty of Science, University of Ibadan, Nigeria; 3 POPs Environmental Consulting, Lindenfirststr. 23, 73527 Schwäbisch Gmünd, Germany

**Keywords:** polymers, plastics, plastic inventory, Nigeria, marine litter, pollution, waste management

## Abstract

**Background.:**

Plastic is a waste stream with recycling and recovery potential. However, the recycling rates for plastic in African countries are low. Furthermore, use and production of virgin plastics are increasing. Therefore, a high proportion of plastic waste is being disposed of in landfills and dumpsites. Plastic serves as fuel for open burning at landfills/dumpsites with associated releases and constitutes a large fraction of marine litter, making it a major and growing global pollution concern.

**Objectives.:**

This study aims to develop an initial inventory of plastics in Nigeria towards the development of an effective plastics management frame.

**Methods.:**

A search was conducted of the recent literature and the United Nations (UN) Comtrade database using harmonized system (HS) codes for import data of various polymer categories and major product categories containing plastic. Algebraic expressions were developed for calculation of the share of plastic in these products.

**Results.:**

Approximately 14,200,000 tons of plastics in primary form were imported into Nigeria between 1996–2014. Approximately 3,420,000 tons total plastic were imported in the form of products and approximately 5,545,700 tons were imported as product components. Approximately 194,000 tons of plastic toys were imported over a six-year period.

**Discussion.:**

The total amount of plastics imported in primary form and as products equals 17,620,000 tons. The total volume of imported plastic, newly produced plastic and plastic components going into the technosphere was 23,400,000 tons. The huge amount of plastic and other polymers entering the technosphere in Nigeria has important implications for marine litter, pollution, waste management and resource recovery.

**Conclusions.:**

A huge volume of plastics has entered the Nigerian technosphere, with less than 12% of the resulting waste entering the recycling stream. There is a need for sustainable management of this crucial waste and resource category. Potential mitigating strategies include waste plastic reuse, recycling, waste conversion to energy, and appropriate plastic control policy frameworks.

**Competing Interests.:**

The authors declare no competing financial interests.

## Introduction

Plastics are materials or products made from a wide range of polymers of high molecular mass. They have broad applications in time and space due to their durability, ease of production, low cost and relatively light weight. Recent decades have witnessed a tremendous increase in plastic used in a wide range of products. Global production of this commodity increased from 1.5 million tons (Mt) per year in 1950 to 245 Mt in 2008, and it has been projected that global plastic production could triple by 2050.^1^ Its use has increased twentyfold in the past half-century and is expected to double again in the next 20 years.^2^ Plastic is increasingly replacing metals, glass, ceramics and wood in many products. Plastic packaging materials are now employed in the food, beverage and other fast-moving consumer goods industries.^3^ However at their end-of-life, plastics present solid waste management challenges. The challenge of plastic waste management, particularly recycling, is a global issue, especially in African countries where robust waste management systems are lacking.

Plastic is a waste stream with recycling and recovery potential.^4^ However, the rate of recycling is not keeping up with the rate at which virgin plastics are being produced and a higher proportion of plastics is being disposed of in landfills and dumpsites than ever before.^5^ Very few polymers are recycled on a large scale.^6^ The recycling and recovery rates for plastic in African countries are low. Furthermore, waste plastics may contain hazardous substances, thereby limiting the scope of recycling.^7^,^8^ Emission of volatile organic compounds during recycling may pose acute and chronic health risks in recycling workshops.^9^ A broad range of plastics contain endocrine-disrupting chemicals such as phthalates or brominated or chlorinated flame retardants.^10–12^ Such contaminants are not commonly removed in recycling of household plastics.^13^

Marine litter has become a global and regional issue affecting the quality of marine and coastal environments.^14^ It constitutes a major threat to marine animals.^15^,^16^ The volume of marine litter is continually growing. Recent studies have shown that litter quantities on some beaches will exceed present levels by 250 times in 10 years.^17^ The majority of litter is from land-based sources. Plastic constitutes a larger fraction of marine litter and has become a major and growing global pollution concern.^18^ Globally, 6.1 billion tons of plastics have been produced, and it has been estimated that about 10% of this will be deposited long-term in the world's oceans.^19^ It is estimated that 275 Mt of plastic waste was generated in 192 coastal countries in 2010, with 4.8 to 12.7 Mt entering the ocean, of which 0.25 to 1.00 Mt from Nigeria was available to enter the ocean in 2010.^20^ This deposition becomes a sink for microplastics in the Arctic sea ice, and can persist in the marine environment for hundreds of years.^1^,^21^ Marine litter is increasing on the deep Arctic seafloor and spreading to the north.^22^,^23^ The regional contribution to the increasing volumes of marine litter along with pollution on land and open burning is an issue that needs to be urgently addressed, particularly in developing countries.

The lack of appropriate solid waste management is a major problem in Africa and other developing countries and a major reason for plastic pollution.^24^,^25^ The region is faced with challenges due to inadequate technological capability and ineffective waste collection systems. There is no source separation of solid waste in Nigeria and therefore a larger fraction of waste plastic and other polymers end up at dumpsites together with other disposed wastes. A preferred management option for plastic is recycling, which is being practiced to an extent in this region. In addition to a limited technical recycling capacity, there is a risk of contamination of recycled plastic by hazardous substances.^8^,^26^ Polymers that do not get into the recycling stream are disposed of through open burning and landfill fires with associated pollution releases. Another share of plastic is dumped in streams and rivers.^25^ Recent studies have shown that rivers are a source of marine litter, and Nigeria ranks 6th in global plastic marine litter release.^27^ Thus, there is an urgent need for more effective management of waste plastics in developing countries such as Nigeria. There is a need for adequate information on plastic/polymer quantities and categories of plastics in Nigeria in order to improve plastic management and to set appropriate and effective policies and incentives.

Although there is growing concern over plastics and related pollution, robust national and regional information is lacking in Africa. A detailed assessment of material/pollutant imports into this region is useful as a baseline for future efforts to reduce pollution levels. Furthermore, the background metric is crucial to accurately measure increases in pollution and as a tool to measure subsequent efforts to reduce pollution levels. Therefore, the present study identifies and synthesizes dispersed data on the import and production of plastic and presents the first national analysis of this data. In addition to compiling information on the import and production of plastic in Nigeria, data is presented concerning plastic imported in products such as cars, washing machines, air conditioning machines, refrigerators, consumer electronics and IT and telecommunication equipment, and toys. In addition, data gaps are identified in our understanding of the overall plastic stock in Nigeria in order to facilitate policy development.

**Abbreviations**
*ABS*: Acrylonitrile butadiene styrene
*EEE*: Electrical electronic equipment
*HS*: Harmonized system
*PVC*: Polyvinyl chloride
*UN*: United Nations
*WEEE*: Waste electrical and electronic equipment

## Methods

The present study method involved a search through the United Nations (UN) Comtrade database using harmonized system (HS) codes for import data of various polymer categories and major product categories containing plastic (see Supplemental Material 1). 28,29 The recent literature was also searched to identify products which contain significant amounts of plastic (e.g. plastic in electrical and electronic equipment (EEE) and waste electrical and electronic equipment (WEEE), the transport sector and other applications). The share of plastic in these products was calculated. Data gaps and inconsistencies were identified and an approach for data improvement is described.

For a reasonable estimation of imported plastics, the assessment covered plastics imported as products and as components of products. As there are numerous products containing polymers imported into Nigeria, the estimation process is complex. In order to simplify our approach, mathematical models or algebraic expressions were developed and applied where appropriate and are shown in Table 1. The main products considered to contain considerable amounts of polymers were refrigerators, washing machines, air conditioning units, consumer electronics, IT and telecommunications equipment, and toys. The assessment covered the period from 1996–2014.

**Table 1:** Approaches to Calculate Plastic in Various Applications in Nigeria

**Parameter**	**Approach**	**Equation Number**	**Explanation/Definition of Terms**
***Import of plastics***	*M*_*i*_= *M*_*p1*_ + *M*_*p2*_	1	*M*_*i*_ is the total plastic (not as product components) imported; *M*_*p1*_ is the total amount of plastic imported in primary form; *M*_*p2*_ is the total amount of plastic imported as products.
***Plastic and other polymers imported in products***	*M*_*ic*_ = *M*_*p*_ * *f*_*p*_	2	M_ic_ is the amount of plastic imported as product components; M_p_ is the amount of a particular product imported; f_p_ is the fraction of plastic in a particular product.
***Polymer content of refrigerators***	*M*_*ic*_ = *M*_*p*_ * *0.32*	3	For refrigerators, the average fraction of plastic is approximately 32% (including polyurethane foam).^30^
***Polymer content of air conditioners***	*M*_*ic*_ = *M*_*p*_ * *0.18*	4	The average plastic fraction of air conditioners is 18%.^31^
***Polymer content of washing machines***	*M*_*ic*_ = *M*_*p*_ * *0.25*	5	The average plastic component of washing machines is approximately 25%.^32^
***Total weight of motor vehicles***	*W*_*tv*_ = *A* * *B*	6	Total weight (W_tv_) of motor vehicles imported: A is the number of motor vehicles imported (1980–2010); B, the average weight of a motor vehicle.
***Total amount of plastic in motor vehicles***	*M*_*pv*_ = *A* * *B* * *C*	7	Total amount of plastic (M_pv_) in imported motor vehicles; C is the plastic fraction.
***Total amount of polyurethane foam***	*M*_*pur*_ = *A* * *D*	8	Total amount of polyurethane foam (M_pur_) in imported motor vehicles; D= polyurethane foam content
***Total polymers in motor vehicles***	*T*_*pv*_ = *M*_*pv*_ + *M*_*pur*_	9	T_pv_ is the total polymers in motor vehicles; M_pv_ is the total amount of plastic; M_pur_ is the total amount of polyurethane foam.

## Results

Table 2 presents the quantity of plastics imported into Nigeria between 1996–2014. Table summarizes the various sub-categories of plastics and detailed definitions are provided in Supplemental Material 1. Detailed applications of these plastics are presented in Table 3.

**Table 2:** Import of Various Categories of Plastics in Primary Forms (tons) into Nigeria

**Year**	3901^*^	**3902**	**3903**	**3904**	**3905**	**3906**	**3907**	**3908**	**3909**	**3910**	**3911**	**3912**	**3913**	**3914**
***1996***	118000	68 702	3548	178000	176	1650	35391	452	4087	1555	1908	35327	1510	36
***1997***	446000	42621	62367	94289	417	1807	27698	694	2966	3877	477	6578	12738	127
***1998***	296000	141000	4004	87415	160	10070	45724	128	9671	673	4532	12615	17554	331
***1999***	59914	81724	2822	23862	74	909	64899	213	6437	771	345	2199	1287	21
***2000***	95634	104000	3968	36853	114	2065	30741	204	2351	524	291	1600	900	27
***2001***	119000	117000	9146	37957	7	1383	48741	182	2916	689	325	2109	770	77
***2002***	185000	1280000	4358	46315	n/a	2428	56612	443	2490	978	826	2045	792	135
***2003***	182000	164000	6500	47262	20	4262	59435	884	2640	968	412	3940	575	74
***2006***	332000	278000	11498	71293	766	18802	265000	3622	4244	1138	700	15491	1933	241
***2007***	616000	333000	18766	129000	705	15284	169000	813	4579	1293	751	6832	663	373
***2008***	187000	159000	33464	88982	239	6790	72623	520	2719	1079	559	4083	167	0
***2009***	338000	223000	19038	60172	141	23941	200000	2173	8299	25777	23566	61648	3144	33
***2010***	498000	262000	23674	84160	531	11551	709000	7972	12456	3280	349	173000	5374	342
***2011***	153000	1120000	15325	98644	178	11515	110000	5224	7569	1475	281	7814	616	232
***2012***	150000	212000	18249	103000	213	11555	134000	1945	5118	1825	612	7419	1184	279
***2013***	321000	116000	28235	115000	193	10889	129000	2318	6803	1377	485	8516	1362	237
***2014***	290000	259000	23727	134000	280	6913	153000	1081	6221	1311	266	7690	144	0
**TOTAL**	**4390000**	**4961047**	**288689**	**1440000**	**4214**	**141814**	**2310864**	**28868**	**91566**	**48590**	**36685**	**358906**	**50713**	**2565**

^*^Harmonized System Code categories. See Supplemental Material 1 for definition of codes.

**Table 3:** Classification and Applications of Common Plastics

**Plastic type**	**SPI Code**	**Applications**
***Polyethylene terephthalate***	1	Beverage bottles, medicine jars, peanut butter jars, combs, bean bags, rope, tote bags, carpet, fiberfill material in winter clothing, plastic film, microwavable packaging
***Polyethylene***		Wide range of inexpensive uses including supermarket bags, plastic bottles
***High-density polyethylene***	2	Containers for milk, motor oil, shampoos and conditioners, soap bottles, detergents, and bleach, toys, plastic crates, plastic lumber, fencing, moulded plastic cases, buckets, rigid pipes, plant pots, plastic woods, garden furniture, wheeled refuse bins, compost containers
***Polyester***		Fibers, textiles
***Polyvinyl chloride (PVC)***	3	Pipes and tiles, flooring, mobile home skirting, and other industrial-grade items, credit cards, window and door frames, wire and cable sheathing, synthetic leather products, shower curtains
***Low-density polyethylene***	4	Plastic cling wrap, sandwich bags, squeezable bottles, plastic grocery bags, garbage cans, lumber, furniture, films, fertilizer bags, refuse sacks, bubble wrap, irrigation pipes, some bottle tops
***Polyvinylidene chloride***		Food packaging, such as plastic cling wrap
***Polypropylene***	5	Plastic diapers, Tupperware, margarine containers, yogurt boxes, syrup bottles, prescription bottles, some stadium cups, ice scrapers, rakes, battery cables, plastic bottle caps, potato crisp bags, biscuit wrappers, crates, plant pots, drinking straws, hinged lunch boxes, refrigerated containers, fabric/carpet fibers, heavy duty bags/tarpaulins, car fenders (bumpers), plastic pressure pipe systems
***Polystyrene***	6	Disposable coffee cups, plastic food boxes, plastic cutlery, packing foam, and packing peanuts, insulation, license plate frames, rulers, yogurt containers, egg boxes, video cases, fast food trays, seed trays, coat hangers, low cost brittle toys, plastic tableware
***High impact polystyrene***		Refrigerator liners, food packaging, vending cups
***Polyamides (nylons)***		Fibers, toothbrush bristles, tubing, fishing line, low-strength machine parts such as engine parts or gun frames
***Acrylonitrile butadiene styrene (ABS)***		Electronic equipment cases (e.g. computer monitors, printers, keyboards), drainage pipe
***Polyethylene/ABS***		A slippery blend of polyethylene and ABS used in low-duty dry bearings
***Polycarbonate***		Compact discs, eyeglasses, riot shields, security windows, traffic lights, lenses
***Polycarbonate/ABS***		A blend of polycarbonate and ABS that creates a stronger plastic used in car interior and exterior parts and mobile phone bodies
***Polyurethane***		Cushioning foams, thermal insulation foams, surface coatings, printing rollers (currently sixth or seventh most commonly used plastic material, for instance the most commonly used plastic in cars)

Source: Adapted from Quality Logo Products; Andrady and Neal Ryedale District Council.^33–35^

Abbreviations: ABS, acrylonitrile butadiene styrene; PVC, polyvinyl chloride; SPI, Society of the Plastics Industry.^36^

Table 4 presents information on the various categories of plastics imported as products into Nigeria.

**Table 4:** Amount (tons) of Plastics Imported as Products into Nigeria

**Year**	3915^*^	**3916**	**3917**	**3918**	**3919**	**3920**	**3921**	**3922**	**3923**	**3924**	**3925**	**3926**
***1996***	1590	656	8830	1940	23465	19987	6871	238	16119	2509	116	4420
***1997***	2330	446	12000	6958	2379	40457	3737	185	3072	486	235	5912
***1998***	764	1190	3640	8629	4446	10302	7403	9	13917	33197	133	11113
***1999***	4221	440	2469	2374	3891	96306	3666	258	2098	1528	90	2431
***2000***	4490	507	1420	6044	2287	6632	4502	336	2122	549	116	1369
***2001***	2090	166	2430	2550	4718	11950	3614	831	11295	1782	53	1573
***2002***	947	393	4110	8676	5097	82975	11420	2151	7231	9603	155	3865
***2003***	1350	221	4500	7997	49541	18576	16899	870	13100	5565	549	4812
***2006***	1150	2880	9170	3799	27623	94764	12218	3525	24069	1667	486	16707
***2007***	9800	409	10800	3614	3503	46208	6347	71	4244	393	714	1249
***2008***	20600	834	9230	10937	3706	32043	9717	584	2813	813	112	3238
***2009***	18400	6290	55700	28681	23595	127000	69167	9781	54617	5432	468	39572
***2010***	13600	8390	54900	56297	84687	373000	129000	369	19595	8338	5226	163000
***2011***	5260	2200	47900	18270	22031	140000	46063	411	14974	4195	565	22317
***2012***	6240	2610	29700	27078	18312	53072	36015	1192	11877	2041	448	24114
***2013***	9110	3090	30700	40169	24547	63392	49925	622	10655	3439	411	17862
***2014***	77600	1820	12700	11552	6226	40671	15534	307	7905	753	579	2035
**TOTAL**	**180000**	**32500**	**300000**	**245565**	**310000**	**1260000**	**432000**	**21700**	**220000**	**82300**	**10500**	**326000**

^*^Harmonized System Code categories. See Supplemental Material 1 for definition of codes.

Table 5 presents data across various categories of refrigerators, air conditioning machines, washing machines and plastic toys imported to Nigeria between 1996–2014. The total amount of refrigerators imported was approximately 456,000 tons. The plastic share of refrigerators is 20%, with about 12% of polyurethane foam, and the total polymer content is 32%.^30^ This amounts to 145,920 tons of plastic and polyurethane foam.

**Table 5:** Refrigerators, Air conditioning Machines, Washing Machines and Plastic Toys Imported into Nigeria (1996–2014)

**Commodity Harmonized System Code**	**Appliance Description**	**Amount (tons)**
***841810***	Combined refrigerator-freezers, fitted with separate external doors	163,000
***841821***	Refrigerators of household type, compression type	92,100
***841822***	Refrigerators of household type, absorption type, electrical	356
***841829***	Other refrigerators of household type	45,670
***841830***	Freezers of the chest type, not exceeding 900 liters capacity	146,000
***841840***	Freezers of the upright type, not exceeding 900 liters capacity	8,740
**TOTAL REFRIGERATORS/FREEZERS**		**456,000**
***841510***	Air Conditioning Units	448,330
^*^8450	Washing machines	109,204
^**^9503	Plastic Toys	387,986

Source: Adapted from UN Comtrade^28^

^*^See Supplemental Material 2 for details

^**^See Supplemental Material 3 for details

The amount of air conditioning units imported in Nigeria was 224,165 tons. From equation (4), the plastic component is M_pa_ = 224,165 × 0.18 = 403,50 tons.

The total amount of washing machines imported equalled 109,204 tons.

The plastic fraction was calculated according to Equation 6:

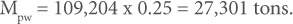



Plastic toys (HS code 950300) include tricycles, scooters, pedal cars and similar wheeled toys, doll carriages, dolls, reduced-size (scale) models and similar recreational models, working or not, puzzles, and other toys. The amount was 193,993 tons. The database did not include earlier data, resulting in an underestimation of this category.

The total amount of polymers imported as components of motor vehicles in Nigeria is shown in Table 6. The total amount of polymers (plastic + polyurethane foam) is estimated to be 2,932,128 tons, including 2,531,128 tons plastic and 401,000 tons of polyurethane foam.

**Table 6:** Amount (tons) of Polymers Imported as Motor Vehicle Components into Nigeria

**Vehicle**	**Number imported (1980–2010)^a^**	**Average weight (kg)**	**Total weight (tons)**	**Plastic fraction (%)**	**Total amount of plastic (tons)**	**^a^Polyurethane foam content (kg)**	**Total amount of polyurethane foam (tons)**
	**(A)**	**(B)**	**(A × B)**	**(C)**	**(A × B × C)**	**(D)**	**(A × D)**
***Cars***	13,000,000	1250^b^	16,250,000	9.1^b^	1,478,750	16	208,000
***Buses***	6,000,000	1850^c^	11,100,000	9.1^b^	1,010,100	32	190,000
***Trucks***	190,000	2649^d^	503,310	8.4^e^	42,278	16	3,000
**TOTAL**					2,531,128		401,000

^a^Babayemi et al;^26^

^b^Gerrard and Kandlikar;^37^

^c^Van Vliet XL Group;^38^

^d^Chevrolet;^39^

^e^Economics and Statistics Department^40^

**Table 7.** Total Amount of Plastics from Various Sources Imported into Nigeria

**Plastic Source**	**Amount (tons)**
Imported plastic (in primary form and as products)	17,620,000
Plastic components of refrigerators	145,920
Plastic components of air conditioning equipment	40,350
Plastic components of laundry washing machines	27,301
Plastic components of motor vehicles	2,932,128
Categories 3 and 4 of European Union EEE/WEEE classification	2,400,000
Toys	193,993
**TOTAL**	**23,400,000**

The amount of plastic contribution from various sources is summarised in Table 10. The total amount of plastic and polymers imported in primary form was 14,200,000 tons, the total amount of plastic and other polymers in major imported products was 3,420,000 tons, and the total amount of plastics imported in primary form and as products was 17,620,000 tons. Therefore, the total volume of imported plastic, newly produced plastic and plastic product components going into the technosphere from 1996–2014 was estimated to be 23,400,000 tons.

## Discussion

The present assessment identified the contribution of imported plastics across a wide range of products, including plastics imported as products and as components of products to estimate the amount of plastic entering the technosphere in Nigeria during the study period.

### Import of plastics in primary form

Approximately 4,390,000 tons of ethylene polymers (polyethylene, ethylene-vinyl acetate copolymers and other polymers of ethylene) were imported into Nigeria between 1996–2014. This means that about 30% of plastic was imported in its primary form, accounting for 19% of total plastics imported during this time period. These plastics are used in the production of supermarket bags, plastic bottles, medicine jars, combs, rope, carpet, plastic film, garbage cans, furniture, fertilizer bags, refuse sacks, irrigation pipes and some bottle tops. These products are abundantly present in Nigeria. The data from other countries outside of Africa also demonstrate the economic importance, wide application and high demand for this type of polymer. The total polyethylene demand in Asia in the year 2017 was around 41.5 Mt, with China accounting for about two-thirds (approximately 27 Mt) of total demand.^41^ Polyolefins make up 60% of the total plastics consumption in India, with packaging being the main plastics consuming sector (42% of total consumption), followed by consumer products and the construction industry.^42^ In 2016, China imported 7.3 Mt of waste plastics, of which polyethylene amounted to 2.53 Mt.^41^ However, this plastic import ceased in January 2018 due to a policy change in China, which may have impacted the plastic recycling sector in Africa/Nigeria as well.

Polymers of propylene or other olefins are also imported in primary form in Nigeria, accounting for approximately 4,961,000 tons, similar to the import rate of polymers of ethylene. This primary form of polymers of propylene includes polyisobutylene, propylene copolymers and other polymers of propylene or olefins. Products made from these polymers include plastic diapers, margarine containers, yogurt boxes, syrup bottles, rakes, battery cables, plastic bottle caps, biscuit wrappers, crates, drinking straws, etc. These products are in high demand and are increasingly used, and consequently large quantities have been dumped as waste. It is very rare to see anyone in Nigeria picking up or collecting drinking straws, biscuit wrappers, etc, for any purpose other than dumping with general wastes which are not sorted prior to incineration.^24^

Approximately 288,700 tons of polymers of styrene were imported into Nigeria from 1996–2014. These polymers include expansible polystyrene, styrene-Acrylonitrile copolymers, Acrylonitrile butadiene styrene copolymers, and other polystyrenes in primary forms. Polystyrene has generally been used in the production of disposable cups, plastic food boxes, insulation, license plate frames, rulers, egg boxes, video cases, seed trays, coat hangers, brittle toys, etc. Acrylonitrile butadiene styrene (ABS) is largely applied in electronic equipment (e.g. television sets and computer monitors, printers, keyboards) and drainage pipes. In 2015, the Indian styrenics market amounted to 526 kilotons (kt) (polystyrene amounting to 241 kt; ABS, 183 kt; and (styrene acrylonitrile, 102 kt).^43^ In comparison, the historical import of 288,700 tons of styrenes in Nigeria refers to styrenes imported in primary form. In addition, these polymers were not imported in millions of tons as polymers of ethylene and propylene, perhaps because electronic devices such as television sets, computers, printers and keyboards, which are the main applications of ABS are being imported rather than being produced in Nigeria. Recent assessments have shown that products at their end-of-life containing these polymers were abundantly present in the country.^8^

Approximately 1,440,000 tons of vinyl chloride polymers in primary form were imported into Nigeria from 1996–2014. This includes polyvinyl chloride not mixed with any other substances, plasticized and non-plasticized polyvinyl chloride, vinyl chloride-vinyl acetate copolymers, vinylidene chloride, and other polymers of vinyl chloride or of other halogenated olefins (polytetrafluoroethylene). These plastics have applications in pipes, tiles, flooring, credit cards, window and door frames, wire and cable sheathing, synthetic leather products, shower curtains and food packaging. Asbestos and wooden ceilings are now largely being replaced with polyvinyl chloride (PVC), resulting in a large material flow of this product, at over a million tons. The import of vinyl acetate polymers appears to be relatively low, at approximately 4200 tons. Applications include production of adhesives such as polyvinyl acetate emulsions, production of copolymers in textile and adhesive resins, printing inks, paperboard coatings, etc. In comparison with the Asia-Pacific region, in 2014, the consumption in Taiwan for polyvinyl polymer was 5,663,000 tons; India, 2,430,000 tons; South Korea, 1,477,000 tons; Japan, 1,063,000 tons; Thailand, 515,000 tons; and Malaysia, 280,000 tons.^44^ Globally, the plasticizers market was 8.4 million tons in 2015, with a large share used in soft PVC, requiring up to 50% plasticizers.^45^

Amino resins, phenolics and polyurethanes are another category of plastics which are imported, approximately 91,600 tons. These polymers include urea and thiourea resins, melamine resins. Polyurethane is applied largely in cushioning foams, thermal insulation foams and surface coatings. High volumes of polyurethane foams are used in motor vehicles (cars, buses, and trucks), refrigerators, freezers and acoustics.^25^ Similar import volumes have been reported in other countries. For example, from 2014 to 2016, India imported approximately 108,000 tons of polyurethane.^46^

Overall, approximately 14,200,000 tons of plastics were imported into Nigeria from 1996–2014. This indicates that activities related to manufacturing of products from plastics are abundant in Nigeria.

### Plastics imported as products

The total amount of plastics imported as products between 1996–2014 was approximately 3,420,000 tons. This represents about one-quarter of plastics imported in primary form. This shows that plastic products are generally produced from imported primary plastics in Nigeria rather than being imported. The imported products include rods, tubes, pipes and hoses, floor coverings (rolls and tiles), wall coverings, ceiling covering, plates, sheets, builders' wares, reservoirs, tanks, articles of apparel and clothing accessories (including gloves, mittens and mitts), fittings for furniture, and ornamental articles.

These products are mainly comprised of construction and household materials, which are now old enough for replacement (import reporting started in 1996, approximately 20 years ago). While reservoirs and tanks at their end-of-life form a considerable fraction of the recycling stream, other products in this category usually end up in dumpsites. When buildings are demolished, concrete, wood and metals are targeted for reuse/recycling, while plastic materials are usually disposed of in dumpsites.

### Plastic consumer product components

There are fractions of plastic which are components of other products. The amount of this plastic constituting product components is significant in the overall volume present in Nigeria. Such products include electrical and electronic equipment like refrigerators, air conditioners, household or laundry washing machines, consumer electronics and IT and telecommunications equipment. In addition, motor vehicles contain a considerable and increasing share of plastic and other polymers. At their end-of-life, these also represent a large waste stream. Plastic from e-waste and light fraction of car shredder residues are a particular challenge in waste management and recycling in Nigeria and in industrial countries as well.^8^,^47^,^48–50^

Toys are largely made of plastics and are an important contributor to total household plastics and of concern with respect to human exposures, in particular for vulnerable infants and children. These major consumer products were therefore also assessed for their contribution to the total plastic stock in Nigeria.

#### Refrigerators

The import of refrigerators (new and second-hand) into Nigeria is continually increasing. A total of 456,000 tons of refrigerators were imported during the study period, containing 145,920 tons of plastic and polyurethane foam. The amount present in Nigeria might be higher than what has been reported. There are local assemblers/manufacturers in the Nigerian EEE sector who often import parts for assembly into Nigeria. According to the Nigeria e-waste country assessment, the volume of assembled/manufactured refrigerators in the country between 2001 and 2005 alone was 124,781 tons.^50^ Therefore, the amount of polymers present in refrigerators in Nigeria is considerable larger than this estimate. The prevalence of refrigerators in urban households is 100% (with average number of 1.27 per household), and approximately 20% in rural areas (with average number of 0.23 per household). 50

#### Air conditioning units

Air conditioning units (HS code 841510) are comprised of a motor-driven fan and elements for changing the temperature and humidity, and are variously fixed to a window, wall, ceiling or floor, either as a self-contained or “split-system”. A total of 224,165 tons of air conditioning units were imported into Nigeria during the study period (1996–2014) containing 40,350 tons of plastics. Generally, air conditioners contain a variety of plastic types, including ABS, PVC, polystyrene and insulating polyurethane foams.^28^

#### Consumer electronics and IT and telecommunications equipment

Major plastic volumes in EEE/WEEE stem from consumer electronics and IT and telecommunications equipment. The IT and telecommunications equipment (European Union WEEE category 3) include fax machines, landline and mobile phones, laptops, personal computers, monitors (cathode ray tubes and flat panels), modems, printers, scanners, copy machines, uninterruptible power supplies (an electrical apparatus that provides emergency power to a load when the input power source or mains power fails) and inverters. Consumer equipment (European Union WEEE category 4) includes televisions (cathode ray tubes and flat panel), alarm clocks, cameras, DVD players, game consoles, MP3 players, radios, stereos, and sewing machines.^51^ Based on the Nigerian EEE/WEEE inventory, the total share of plastic in these two major EEE categories has been estimated by Babayemi et al. to be 2.4 million tons between 2000–2010 in Nigeria.^8^

#### Motor vehicles

A total of 224,165 tons of polymers (plastic + polyurethane foam) are estimated to have been imported into Nigeria from 1980–2010. Vehicles are another major source of plastics and polymer imports into Nigeria. Plastic car body parts include spoilers and bumpers, instrument panels and headlights, side trim and interior trim, seats and airbags, carpets, tires, seals and gaskets, fan belts, gearbox mountings, engine covers, etc.^52^ The main polymers used in their manufacture include polypropylene, polyurethanes, nylon, polyvinyl chloride, ABS, polyethylenes, polycarbonate, and polyvinyl butyral.^53^ The average percentage of plastic in cars and buses is 9.1% and 8.4% in trucks, respectively, as used for estimations in this study. 53,40 The plastic share in vehicles is increasing. Recent models of cars may have up to 15% plastic share of their total weight.^52^ These plastic/polymers end up in the light shredder fraction in the management of end-of-life vehicles and are a waste management challenge.^13^

#### Toys

Between 2009–2014 in Nigeria, 193,993 tons of toys were imported. Toys are inexpensive products and the global market is dynamic, with a high rate of growth. Toys are often made from heterogeneous materials and may contain chemical components other than polymers.^54^ Toys can contain hazardous chemicals such as flame retardants, including persistent organic pollutants (POPs) from recycling or softeners as additives and may be contaminated with heavy metals.^55–57^ This may pose recycling challenges, especially for developing countries.

### Total amountå of plastic in the Nigeria technosphere

The total amount of plastics imported into Nigeria from 1996–2014 (including plastics from motor vehicles from 1980–2010) was estimated to be 23,400,000 tons (23.4 Mt). Plastics produced in Nigeria are manufactured from imported raw materials or from recycled waste and therefore may not be counted as an additional contribution source to the volume of plastic in Nigeria. The large volume of plastic and polymers entering the technosphere in Nigeria has important implications for marine litter, pollution, waste management and resource recovery. It is crucial to account for the fate of imported plastics in Nigeria through all of the life cycle stages: import, storage, use, recycling, burning, landfill, and export.

### Plastic industry in Nigeria and initial attempts at recycling

The Nigerian plastics and packaging sector started with about 50 plastics companies in the 1960s and had grown to over 3,000 companies in 2013 with a production capacity of over 100,000 tons per year.^58^ There is a growing market for plastic products in Nigeria driving this development. Products from recycled plastic are now abundant in the country, including furniture, packaging materials (nylon bags), footwear, hangers, boxes, foot mats, waste bins, and water containers, among others.

Waste plastics that are generally recycled include used plastic furniture, reservoirs, bowls and buckets. The fraction of other categories of recycled plastics depends on circumstances. For instance, waste plastic water bottles are collected for recycling during ceremonies or in restaurants. However, plastic bottles used in households usually end up at dumpsites with general waste, because wastes are not typically separated at the source. Other plastics which are not generally sorted for recycling include waste nylons, drinking straws, plastic labels, used plastic pens, etc. This contributes greatly to environmental pollution. Overall, the recycling rate of total plastic is low, less than 12% annually.^59^

Plastic in electronics and vehicles are high value plastics (e.g. ABS, high impact polystyrene) with a high recovery value if separated appropriately. However, these polymers often contain flame retardants and some of are listed as POPs in the Stockholm Convention.^60^,^61^

### Plastic waste in Nigeria

In Nigeria, less than 12% of plastic waste is recycled. There is no current capacity for energy recovery in cement kilns or incinerators with heat recovery. About 80% of plastic waste goes to landfills and dump sites.^59^ Other disposal options include open burning and landfill fires, resulting in air pollution. In this initial inventory, the amount of plastic disposed of by open burning was not quantified; however, it may be similar to the estimate in Babayemi et al. for plastic from e-waste.^8^,^25^

Globally, as of 2015, the amount of plastic waste generated was 6300 Mt, and of this, 9% was recycled, 12% incinerated, and 79% accumulated in landfills.^62^ In the European Union, around 25 Mt of plastic waste was generated in 2008.^1^ However, industrialized countries with separation schemes and in place legislation have high recycling and recovery rates. In these countries, plastics may be recycled or shipped to China or India for recycling. In India, 47% of the total plastic waste generated is recycled.^42^

### Study data

The data compiled for the present study was evaluated for reliability, gaps and inconsistency. The accessed UN Comtrade database (considered the most comprehensive with more than 1 billion records with continuous updates) contained detailed import and export trade data of about 200 countries from 1962 to the most recent year.^28^ Before data received from national authorities were added to the database, they were usually standardised by the UN Statistics Division. However, the trade of a country could be understated due to unavailability of some country data, because some countries do not necessarily report their trade data for each and every year. This may therefore create gaps for a specific year with no available data.

UN Comtrade receives data from Nigeria through the National Bureau of Statistics, Federal Republic of Nigeria, Food and Agriculture Organization of the United Nations (FAO), and the International Trade Commission, etc.^63^

### Gaps and inconsistency of import data in UN Comtrade

Data for the years 2004 and 2005 were missing for all imported plastics in primary form and as products. In other cases, data for one or more years were missing for a category of products. This does not mean that plastics were not imported during this period (zero was recorded where there was no import), but that the database did not receive information for the respective period/year and HS category. Due to this missing data, the actual amount of plastic imported is somewhat higher than reported in this paper.

In addition, the data revealed some particularly high import rates in 2009 and 2010. These years were unique in Nigeria's economy, including for import of goods. Generally, import of all goods in Nigeria reached an all-time peak in 2010, and import partners included China, Belgium, Netherlands and the United States of America.^64^ Import of finished goods was also highest in 2010.^65^ Import of construction materials was generally high in Nigeria in 2010, and imported polymer materials for construction include roofing sheets, PVC tanks, PVC tiles, and PVC plumbing materials.^66^ In addition, there was a peak in the import of used electrical electronic equipment around 2010 in Nigeria after China strongly reduced importation of WEEE around 2006.^8^ Other factors responsible for high import rates may include an increase in population from 120 million in 2000 to 160 million in 2010. Apart from 2010, there were some high import rates within specific categories for various years. It may be that relevant industries or manufacturers had special orders resulting in higher volumes over those periods. However, it was not possible to trace individual data and industries for confirmation.

The import data for a country are usually recorded with relative accuracy as imports generate tariff revenues. Therefore, the higher/lower data points were considered to be accurate in the Comtrade database.

### Plastic fractions not fully addressed in this study

Only major consumer products containing plastic were included in the current analysis. In terms of EEE/WEEE, only category 3 (IT and telecommunications equipment), category 4 (consumer equipment), category 1 (white goods) and air conditioners were considered. Plastic in small household appliances as well as packaging materials of imported products such as polystyrene packaging or polymer wrapping were not included due to the lack of data. While this approach underestimates the total volume of plastic imported via EEE, the major plastic imports were covered in the present study.

### Selected time frame and material flow analysis

The present study covered the period from 1996–2014 for the compilation of plastic import data. While plastics were imported before 1996, this time period is most relevant due to the increasing use of plastic over the last 20 years. The plastic imported over the last two decades has entered the technosphere, while plastic imported before 1996 has generally been disposed of in landfills and dumpsites over the last two decades. A considerable share of the plastics imported since 1996 have reached end of life (EoL) and been disposed of or subject to open burning, including plastic used for disposable goods like packaging or plastic bags. For other plastics such as water pipes or plastics used for window frames or other construction, the largest share is still in use. For plastics in consumer goods such as electronics or vehicles, their status depends on particular product life spans. For an assessment of the stocks and flows of plastic, a material flow analysis for individual products is needed, which is beyond the scope of the present study which compiled primary data on plastic imports to Nigeria. Preliminary material flows have been established by our previous research on brominated POPs in plastic from selected WEEE fractions and in polyurethane foam in vehicles produced between 1970–2004, establishing initial material flow data for Nigerian plastic fractions.^8^,^25^ Further study is needed to address the previously mentioned data gaps and to improve available data.

## Conclusions

More than 23,400,000 tons of plastics entered the Nigerian technosphere between 1996–2014, with less than 12% of the resulting waste in the recycling stream. Considering the risks this volume presents to global and local environments and human health, there is the need for sustainable management of this important waste and resource category.^67^ Potential mitigating strategies may include waste plastic reuse, recycling, waste conversion to energy, and appropriate plastic control policy frameworks. Policies should address the waste hierarchy with an emphasis on waste reduction and recycling. Energy recovery using cement kilns is a promising mitigation solution, as cement kilns in Nigeria do not at present use any secondary fuel.

The present study demonstrated a novel application of international trade data to the estimation of plastic volume flow and associated pollution potential in a developing country. The connection of international trade and inventory data and related waste/pollution potential is a powerful tool that can be used to develop countermeasures and improve prevention and management programs. Furthermore, as there was no previously established methodology for this assessment in Nigeria or the region of Africa as a whole, the approach in this study may serve as a model for similar studies in other developing countries. The data obtained in this study can serve as basis for policies to improve plastic waste management in Nigeria and for future studies refining the dataset and more detailed material and substance flow analyses.
