# Supplementary material for: Initial Inventory of Plastics Imports in Nigeria as a Basis for More Sustainable Management Policies
Source: J Health Pollut. 2018 Jun 11;8(18):180601. doi: 10.5696/22156-9614-8.18.1 (PMC6239059; doi:10.5696/22156-9614-8.18.1)
Supplement: Supplementary file 1 [file i2156-9614-8-18-1.s1.docx]

**Supplemental Material 1**

**Harmonized System (HS) Code Classification of Plastics**

| **Main Polymer Category** | **Code** | **Sub-categories** | **Code** |
| --- | --- | --- | --- |
| Polymers of ethylene, in primary form | 3901 | Polyethylene with a specific gravity of less than 0.94 | 390110 |
|  |  | Polyethylene with a specific gravity of 0.94 or greater | 390120 |
|  |  | Ethylene-vinyl acetate copolymers | 390130 |
|  |  | Other polymers of ethylene, in primary form | 390190 |
| Polymers of propylene or other olefins, in primary form | 3902 | Polypropylene in primary form | 390210 |
|  |  | Polyisobutylene in primary form | 390220 |
|  |  | Propylene copolymers in primary form | 390230 |
|  |  | Other polymers of propylene or other olefins | 390290 |
| Polymers of styrene, in primary form | 3903 | Expansible polystyrene in primary form | 390311 |
|  |  | Other Polystyrene in primary form | 390319 |
|  |  | Styrene-acrylonitrile copolymers in primary form | 390320 |
|  |  | Acrylonitrile-butadiene-styrene (ABS) copolymers | 390330 |
|  |  | Other polymers of styrene in primary form | 390390 |
| Polymers of vinyl chloride etc., in primary form | 3904 | Polyvinyl chloride, not mixed with any other substances (in primary form) | 390410 |
|  |  | Other polyvinyl chloride (non-plasticised) in primary form | 390421 |
|  |  | Other polyvinyl chloride (plasticised) in primary form | 390422 |
|  |  | Vinyl chloride-vinyl acetate copolymers in primary form | 390430 |
|  |  | Other vinyl chloride copolymers in primary form | 390440 |
|  |  | Vinylidene chloride polymers in primary form | 390450 |
|  |  | Polytetrafluoroethylene in primary form | 390461 |
|  |  | Other fluoro-polymers in primary form | 390469 |
|  |  | Other polymers of vinyl chloride or other halogenated olefins | 390490 |
| Polymers of vinyl acetate and other vinyl polymers, in primary form | 3905 | Other polymers of vinyl acetate | 390519 |
| Acrylic polymers in primary form | 3906 | Polymethyl methacrylate in primary form | 390610 |
|  |  | Other acrylic polymers in primary form | 390690 |
| Polyethers, epoxides and polyesters, primary form | 3907 | Polyacetals in primary form | 390710 |
|  |  | Other polyethers | 390720 |
|  |  | Epoxide resins | 390730 |
|  |  | Polycarbonates in primary form | 390740 |
|  |  | Alkyd resins | 390750 |
|  |  | Polyethylene terephthalate in primary form | 390760 |
|  |  | Other Polyesters (Unsaturated) | 390791 |
|  |  | Halo-isobutene-isoprene Rubber (CIIR) | 390799 |
| Polyamides in primary form | 3908 | Polyamide -6, -11, -12, -6, 6 -6, 9, -6, 10, -6, 12 | 390810 |
|  |  | Other polyamides | 390890 |
| Amino resins, phenolics and polyurethanes, in primary form | 3909 | Amino-resins; urea and thiourea resins, in primary form | 390910 |
|  |  | Amino-resins; melamine resins, in primary form | 390920 |
|  |  | Amino-resins not elsewhere classified in heading no. 3909, in primary form | 390930 |
|  |  | Polyurethanes in primary form | 390950 |
| Silicones, in primary form | 3910 | Silicones in primary form | 391000 |
| Petro resins, polysulfides etc. note elsewhere specified or included (nesoi), in primary form | 3911 | Petroleum resins, coumarone, indene or coumarone-indene resins and polyterpenes in primary form | 391110 |
|  |  | Polysulphides, polysulphones and similar products of chemical synthesis not elsewhere classified in chapter 39 in primary form | 391190 |
| Cellulose and chemical deriv. nesoi, in primary form | 3912 | Cellulose acetates, non-plasticised, in primary form | 391211 |
|  |  | Cellulose nitrates (including collodions); in primary form | 391220 |
|  |  | Cellulose ethers; carboxymethylcellulose and its salts, in primary form | 391231 |
|  |  | Cellulose ethers; (other than carboxymethylcellulose and its salts), in primary form | 391239 |
|  |  | Cellulose and its chemical derivatives; not elsewhere classified in item no. 3912, in primary form | 391290 |
| Natural (inc. modified) polymers nesoi, in primary form | 3913 | Polymers, natural; alginic acid, its salts and esters, in primary form | 391310 |
|  |  | Polymers, natural and modified natural, in primary form (excluding alginic acid, its salts and esters) | 391390 |
| Ion-exchangers based on plastics, in primary form | 3914 | Ion-exchangers; based on polymers of heading no. 3901 to 3913, in primary form | 391400 |
| Plastic waste, parings and scraps | 3915 | Ethylene polymers; waste, parings and scrap | 391510 |
|  |  | Styrene polymers; waste, parings and scrap | 391520 |
|  |  | Vinyl chloride polymers; waste, parings and scrap | 391530 |
|  |  | Plastics n.e.c. in heading no. 3915; waste, parings and scrap | 391590 |
| Plastic monofil, cr-sect ovimm, rods, sticks, etc. | 3916 | Ethylene polymers; monofilament, of which any cross-sectional dimension exceeds 1 mm, rods, sticks and profile shapes, whether or not surface-worked but not otherwise worked | 391610 |
|  |  | Vinyl chloride polymers; monofilament, of which any cross-sectional dimension exceeds 1 mm, rods, sticks and profile shapes, whether or not surface-worked but not otherwise worked | 391620 |
|  |  | Plastics; monofilament, of plastics n.e.c. in heading no. 3916, cross-sectional dimension exceeds 1 mm, rods, sticks and profile shapes, whether or not surface-worked but not otherwise worked | 391690 |
| Tubes, pipes, hoses and their fittings | 3917 | Plastics; artificial guts (sausage casings) of hardened protein or of cellulosic materials | 391710 |
|  |  | Plastics; tubes, pipes and hoses thereof, rigid, of polymers of ethylene | 391721 |
|  |  | Plastics; tubes, pipes and hoses thereof, rigid, of polymers of propylene | 391722 |
|  |  | Plastics; tubes, pipes and hoses thereof, rigid, of polymers of vinyl chloride | 391723 |
|  |  | Plastics; tubes, pipes and hoses thereof, rigid, of plastics n.e.c. in heading no. 3917 | 391729 |
|  |  | Plastics; tubes, pipes and hoses thereof, flexible, having a minimum burst pressure of 27.6MPa | 391731 |
|  |  | Plastics; tubes, pipes and hoses thereof, other than those of item no. 3917.31, not reinforced or otherwise combined with other materials, without fittings | 391732 |
|  |  | Plastics; tubes, pipes and hoses thereof, other than those of item no. 3917.31, not reinforced or otherwise combined with other materials, with fittings | 391733 |
|  |  | Plastics; tubes, pipes and hoses thereof, n.e.c. in item no. 3917.30 | 391739 |
|  |  | Plastics; tube, pipe and hose fittings (e.g. joints, elbows, flanges) | 391740 |
| Plastic floor covering (rolls and tiles) and wall covering | 3918 | Floor, wall or ceiling coverings; of polymers of vinyl chloride, whether or not self-adhesive, in rolls or in the form of tiles | 391810 |
|  |  | Floor, wall or ceiling coverings; of plastics (excluding polymers of vinyl chloride), whether or not self-adhesive, in rolls or in the form of tiles | 391890 |
| Plastic self-adhesive plates, sheets, film, etc. | 3919 | Plastics; plates, sheets, film, foil, tape, strip, other flat shapes thereof, self-adhesive, in rolls of a width not exceeding 20 cm | 391910 |
|  |  | Plastics; plates, sheets, film, foil, tape, strip, other flat shapes thereof, self-adhesive, other than in rolls of a width not exceeding 20 cm | 391990 |
| Plates, sheets, film etc. | 3920 | Plastics; plates, sheets, film, foil and strip (not self-adhesive), of polymers of ethylene, non-cellular and not reinforced, laminated, supported or similarly combined with other materials | 392010 |
|  |  | Plastics; of polymers of propylene, plates, sheets, film, foil and strip (not self-adhesive), non-cellular and not reinforced, laminated, supported or similarly combined with other materials | 392020 |
|  |  | Plastics; of polymers of styrene, plates, sheets, film, foil and strip (not self-adhesive), non-cellular and not reinforced, laminated, supported or similarly combined with other materials | 392030 |
|  |  | Plastics; polymers of vinyl chloride, containing by weight not less than 6% of plasticisers; plates, sheets, film, foil and strip (not self-adhesive), non-cellular and not reinforced, laminated, supported or similarly combined with other materials | 392043 |
|  |  | Plastics; of acrylic polymers, polymethyl methacrylate, plates, sheets, film, foil and strip (not self-adhesive), non-cellular and not reinforced, laminated, supported or similarly combined with other materials | 392051 |
|  |  | Plastics; of acrylic polymers (excluding polymethyl methacrylate), plates, sheets, film, foil and strip (not self-adhesive), non-cellular and not reinforced, laminated, supported or similarly combined with other materials | 392059 |
|  |  | Plastics; plates, sheets, film, foil and strip (not self-adhesive), of polycarbonates, non-cellular and not reinforced, laminated, supported or similarly combined with other materials | 392061 |
|  |  | Plastics; plates, sheets, film, foil and strip (not self-adhesive), of poly(ethylene terephthalate), non-cellular and not reinforced, laminated, supported or similarly combined with other materials | 392062 |
|  |  | Plastics; plates, sheets, film, foil and strip (not self-adhesive), of unsaturated polyesters, non-cellular and not reinforced, laminated, supported or similarly combined with other materials | 392063 |
|  |  | Plastics; plates, sheets, film, foil and strip (not self-adhesive), of polyesters n.e.c. in heading no. 3920, non-cellular and not reinforced, laminated, supported or similarly combined with other materials | 392069 |
|  |  | Plastics; plates, sheets, film, foil and strip (not self-adhesive), of regenerated cellulose; non-cellular and not reinforced, laminated, supported or similarly combined with other materials | 392071 |
|  |  | Plastics; plates, sheets, film, foil and strip (not self-adhesive), of cellulose acetate, non-cellular and not reinforced, laminated, supported or similarly combined with other materials | 392073 |
|  |  | Plastics; plates, sheets, film, foil and strip (not self-adhesive), of cellulose derivatives n.e.c. in heading no. 3920, non-cellular and not reinforced, laminated, supported or similarly combined with other materials | 392079 |
|  |  | Plastics; plates, sheets, film, foil and strip (not self-adhesive), of poly(vinyl butyral), non-cellular and not reinforced, laminated, supported or similarly combined with other materials | 392091 |
|  |  | Plastics; plates, sheets, film, foil and strip (not self-adhesive), of polyamides, non-cellular and not reinforced, laminated, supported or similarly combined with other materials | 392092 |
|  |  | Plastics; plates, sheets, film, foil and strip (not self-adhesive), of amino-resins, non-cellular and not reinforced, laminated, supported or similarly combined with other materials | 392093 |
|  |  | Plastics; plates, sheets, film, foil and strip (not self-adhesive), of phenolic resins, non-cellular and not reinforced, laminated, supported or similarly combined with other materials | 392094 |
|  |  | Plastics; plates, sheets, film, foil and strip (not self-adhesive), of plastics n.e.c. in heading no. 3920, non-cellular and not reinforced, laminated, supported or similarly combined with other materials | 392099 |
| Plastic plates, sheets, film, foil and strip nesoi | 3921 | Plastics; plates, sheets, film, foil and strip, of polymers of styrene, cellular | 392111 |
|  |  | Plastics; plates, sheets, film, foil and strip, of polymers of vinyl chloride, cellular | 392112 |
|  |  | Plastics; plates, sheets, film, foil and strip, of polyurethanes, cellular | 392113 |
|  |  | Plastics; plates, sheets, film, foil and strip, of regenerated cellulose, cellular | 392114 |
|  |  | Plastics; plates, sheets, film, foil and strip, of plastics n.e.c. in heading no. 3921, cellular | 392119 |
|  |  | Plastics; plates, sheets, film, foil and strip, other than cellular | 392190 |
| Plastic baths, washbasins, lavatory seats, etc. | 3922 | Plastics; baths, shower-baths, sinks and wash-basins | 392210 |
|  |  | Plastics; lavatory seats and covers | 392220 |
| Plastic containers (boxes, bags etc), closures, etc. | 3923 | Plastics; boxes, cases, crates and similar articles for the conveyance or packing of goods | 392310 |
|  |  | Ethylene polymers; sacks and bags (including cones), for the conveyance or packing of goods | 392321 |
|  |  | Plastics; sacks and bags (including cones), for the conveyance or packing of goods, of plastics other than ethylene polymers | 392329 |
|  |  | Plastics; carboys, bottles, flasks and similar articles, for the conveyance or packing of goods | 392330 |
|  |  | Plastics; articles for the conveyance or packing of goods n.e.c. in heading no. 3923 | 392390 |
| Plastic tableware and other household articles, etc. | 3924 | Plastics; tableware and kitchenware | 392410 |
|  |  | Plastics; household articles and hygienic or toilet articles | 392490 |
| Plastic builders' wares, nesoi | 3925 | Plastics; builders' ware, reservoirs, tanks, vats and similar containers of a capacity exceeding 300 liters | 392510 |
|  |  | Plastics; builders' ware, shutters, blinds (including venetian blinds) and similar articles and parts thereof | 392530 |
| Plastic articles of plastics (inc. polymers and resins) nesoi | 3926 | Plastics; office or school supplies | 392610 |
|  |  | Plastics; articles of apparel and clothing accessories (including gloves, mittens and mitts) | 392620 |
|  |  | Plastics; fittings for furniture, coachwork or the like | 392630 |
|  |  | Plastics; statuettes and other ornamental articles | 392640 |
|  |  | Plastics; other articles not elsewhere classified in chapter 39 | 392690 |
| Toys | 9503 | Tricycles, scooters, pedal cars and similar wheeled toys; doll carriages; dolls; other toys; reduced-size (scale) models and similar recreational models, working or not; puzzles of all kinds | 950300 |

**Supplemental Material 2**

**Amount (tons) of Washing Machines
Imported into Nigeria (1996-2014)**

| **Period** | **HS 845011** | **HS 845012** | **HS 845019** | **HS 845020** | **Total** |
| --- | --- | --- | --- | --- | --- |
| 1996 | 68 | 52 | 13 | 32 | 165 |
| 1997 | 122 | 24 | 31 | 70 | 247 |
| 1998 | 2148 | 28 | 29 | 20 | 2225 |
| 1999 | 96 | 42 | 11 | 6 | 155 |
| 2000 | 136 | 24 | 21 | 248 | 429 |
| 2001 | 333 | 68 | 49 | 24 | 474 |
| 2002 | 395 | 34 | 96 | 9 | 534 |
| 2003 | 382 | 117 | 69 | 39 | 607 |
| 2006 | 6187 | 133 | 46 | 9396 | 15762 |
| 2007 | 1385 | 211 | 208 | 203 | 2007 |
| 2008 | 851 | 108 | 729 | 108 | 1796 |
| 2009 | 41699 | 221 | 1620 | 1259 | 44799 |
| 2010 | 677 | 227 | 2815 | 912 | 4631 |
| 2011 | 836 | 469 | 2266 | 4212 | 7783 |
| 2012 | 648 | 66 | 1049 | 8544 | 10307 |
| 2013 | 971 | 18 | 371 | 9122 | 10482 |
| 2014 | 1702 | 43 | 988 | 4068 | 6801 |

Source: Adapted from UN Comtrade**^28^**

**Supplemental Material 3**

**Amount (tons) of Plastic Toys (HS code 9503)**

**Imported into Nigeria (2009-2014)**

| **Period** | **Amount (tons)** |
| --- | --- |
| 2009 | 67968 |
| 2010 | 86161 |
| 2011 | 13186 |
| 2012 | 10761 |
| 2013 | 13378 |
| 2014 | 2539 |
| Total | 193993 |

Source: Adapted from UN Comtrade**^28^**
